# A Robot-Assisted Cell Manipulation System with an Adaptive Visual Servoing Method

**DOI:** 10.3390/mi7060104

**Published:** 2016-06-20

**Authors:** Yu Xie, Feng Zeng, Wenming Xi, Yunlei Zhou, Houde Liu, Mingliang Chen

**Affiliations:** 1 School of Aerospace Engineering, Xiamen University, Xiamen 361005, China; xieyu@xmu.edu.cn (Y.X.); 19920141152946@stu.xmu.edu.cn (F.Z.); wmxi@xmu.edu.cn (W.X.); 19920141152932@stu.xmu.edu.cn (Y.Z.); 2 Shenzhen Engineering Laboratory of Geometry Measurement Techinology, Graduate School at Shenzhen, Tsinghua University, Shenzhen 518055, China; 3 Key Laboratory of Marine Biogenetic Resources, Third Institute of Oceanography, State Oceanic Administration, Xiamen 361005, China; mlchen_gg@tio.org.cn

**Keywords:** cell manipulation, robotics, adaptive imaging processing, autofocusing

## Abstract

Robot-assisted cell manipulation is gaining attention for its ability in providing high throughput and high precision cell manipulation for the biological industry. This paper presents a visual servo microrobotic system for cell microinjection. We investigated the automatic cell autofocus method that reduced the complexity of the system. Then, we produced an adaptive visual processing algorithm to detect the location of the cell and micropipette toward the uneven illumination problem. Fourteen microinjection experiments were conducted with zebrafish embryos. A 100% success rate was achieved either in autofocus or embryo detection, which verified the robustness of the proposed automatic cell manipulation system.

## 1. Introduction

Microinjecting microliters of genetic material into embryos of model animals is a standard method used for analyzing vertebrate embryonic development and the pathogenic mechanisms of human disease [1,2]. Cell micromanipulation procedure is currently being conducted manually by trained personnel. This requires lengthy training and lack of reproducibility. However, this method cannot meet the demands of the growing development of biological research and the need for testing materials [3]. The integration of robotic technology into biological cell manipulation is an emerging research area that endeavors to improve efficiency, particularly in precision and high throughput aspects.

Recently, several robotic injection prototypes for cell microinjection were reported [4,5,6,7,8,9]. Wang *et al.* used a position control strategy to inject zebrafish embryos, in which a visual servoing method was used to detect the target position of the end-effector, and a PID (proportional-integral-derivative) position control was used for micropipette movement [4]. Position control with force signal feedback was used by Lu *et al.* to inject zebrafish embryos, where a piezoresistive microforce sensor was used to monitor the injection process [5]. A homemade PVDF (Poly vinylidene fluoride) microforce sensor was proposed in [6] to evaluate the haptic force in a cell injection process. Huang *et al.* used vision and force information to determine three-dimensional cell microinjection, and adopted an impedance control method to control the movement of the injector in the *z*-direction [7]. Xie *et al.* employed an explicit force control method to regulate the cell injection force on zebrafish embryos [8,9]. However, two problems remained unsolved. First, these studies focused on the motorized injection strategy and control algorithm, even though vision feedback was adopted in every robotic prototype. Past studies did not focus on visual feedback in these robotic injection systems. The segmentation of the embryo and injection pipette is relatively easy for a fixed image. Since invention of automatic microinjection used for large-scale batch microinjections, one of the main challenges lies in the quality of the real-time images that are affected by the environment (*i.e.*, uneven illumination), cell culture medium or individual cell morphology. Therefore, we focused on adaptive and robust image processing in our visual servo system design.

Second, in addition to automating the embryo injection process, a smart visual servoing structure is able to improve the automation level and simplify the whole manipulation system. For instance, a microscope autofocusing system can bring the samples into focus by using the focus algorithm and motion control. To date, no studies concentrated on the autofocusing method for a robot-assisted zebrafish embryo microinjection.

Section 2 of this paper introduces the architecture of the visual servoing cell microinjection robot system. Section 3 reports on the microscope automatic servoing method used to automate the cell manipulation process. The suitability of different focus criteria was evaluated and a visual servoing motion control method is described for a robotic embryo microinjection system. An adaptive visual processing algorithm developed for real-time cell and micropipette location under different illumination environments is discussed. Finally, Section 4 and Section 5 report the experimental results and discussion of zebrafish embryos microinjection.

## 2. The Automatic Microinjection System

### 2.1. System Configuration

**Figure 1:**
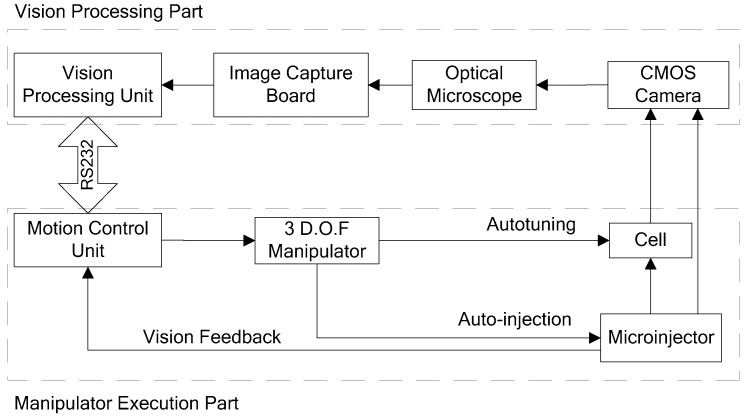
Block diagram of the automatic cell microinjection system.

**Figure 2:**
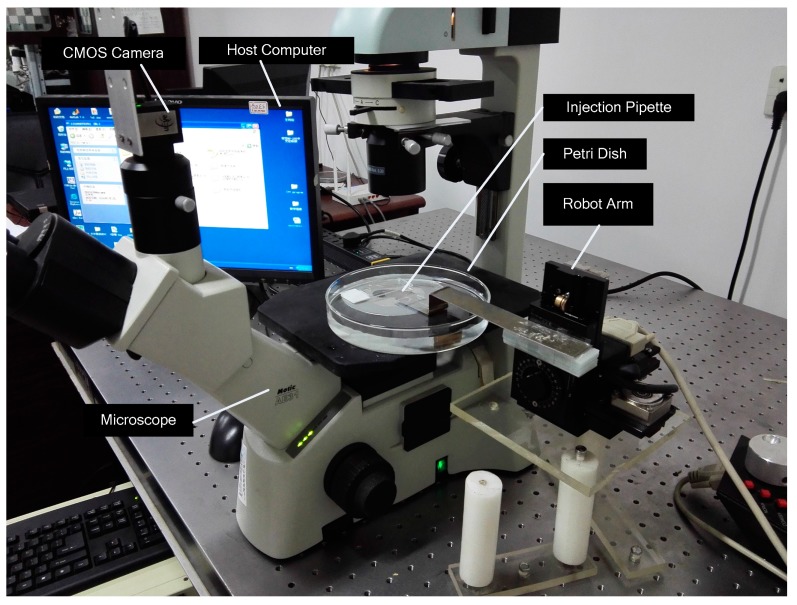
The photograph of the automatic microinjection system.

The visual servo cell microinjection system included: (a) a microscope vision processing part; (b) a micromanipulation part; and (c) an integrated interface software platform for visual servoing. The block diagram of the system is shown in Figure 1. The photograph of the microinjection system is shown in Figure 2.

The visual processing part was responsible for the management of the camera, image acquisition and processing. It included an inverted microscope (model: AE-31, Motic Inc., Wetzlar, Germany) and a CMOS (complementary metal-oxide-semiconductor) camera (model: uEye UI-1540M, IDS Inc., Obersulm, Germany). The microscope had a working distance of 70 mm with a minimum step of 0.2 mm. A CCD (charge-coupled device) adapter of 0.65× and an objective of 4× (N.A. 0.1) were selected to observe the zebrafish embryos. The microscope was working under the bright-field observation mode that provided the necessary optical magnification and illumination levels for proper imaging of the injection area. The CMOS camera was mounted on the microscope with a resolution of 1280 × 1024 pixel, and a 25 fps frame rate was used to acquire the video.

The micromanipulation part managed the motion controlling instructions from the host computer by handling all processing and signal generation to drive the motion devices using the serial and parallel ports. A three-degrees-of-freedom (3-DOF) robotic arm with a 0.04 μm positioning resolution (model: MP-285, Sutter Inc., Novato, CA, USA) was used to conduct the automatic microinjection task.

To determine the visual servoing automatic microinjection, an integrated software platform was necessary to confirm communications among function modules of the image acquisition, image processing, automatic focusing and automatic microinjection. Because the microinjection system was manipulated from a host computer, a graphical user interface (GUI) was also required to enable the interaction between the user and cell micro-world. More details about the software platform are introduced in the next subsection.

### 2.2. Integrated Interface Software Platform for Visual Servoing

The integrated interface software platform for visual servoing control was developed under the Microsoft Visual C++ (6.0) environment to ensure the compatibility and portability among the software modulus and hardware. For image acquisition, the camera Software Development Kit (SDK) used C and a small amount of C++ programming, which is compatible with Microsoft Visual C++. For the image processing algorithm, the Intel OpenCV image processing library was used, which also is compatible with Microsoft Visual C++. The 3-DOF manipulator was controlled by a commercial motion controller and was connected to the host computer by an RS-232 serial port. The Windows API (Application Programming Interface) was used to send and receive the position information of the manipulator.

**Figure 3:**
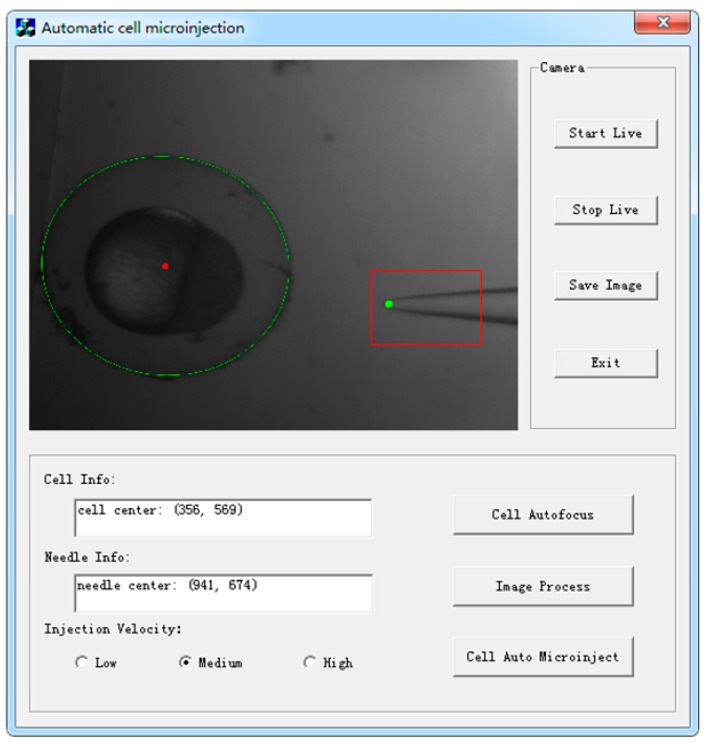
Visual servoing microinjection interactive interface.

A GUI was designed to provide an interactive way to conduct the robot-assisted microinjection procedure. Figure 3 shows the designed visual servoing microinjection interactive interface developed with the MFC (Microsoft Foundation Classes) framework. The functions of the buttons are described in Table 1.

## 3. Visual Servoing Algorithm for Automatic Cell Microinjection

### 3.1. Visual Servoing Algorithm for Automatic Cell Autofocusing

#### 3.1.1. Selection of the Criterion Function

Since the system was used for a large-scale microinjection, speed and reliability were our primary considerations in the development of the autofocus algorithm because they enhance the efficiency and level of automation for the entire system. Criterion functions were studied for the autofocusing of the microscopes and other optical instruments in prior works [10]. Eighteen focus algorithms were compared in [11,12], where variance based focus algorithms were more sensitive to noise while the gradient-based focus algorithms had better performance in sub-sample cases. In our cell injection system, the image for processing is shown in Figure 3. The zebrafish embryo had a symmetric spherical shape and a clear background, with some dampness from the culture liquid. As such, we narrowed the candidate criterion functions to the following: the Brenner gradient, the Tenenbaum gradient and normalized variance algorithms.

The Brenner gradient [13] measured the differences between a pixel value and its neighbor pixel with a vertical or horizontal distance of two pixel positions. The horizontal direction is used in this paper:

(1) $$f(I)=\sum_{x}\sum_{y}\left\{{\left[I(x+2,y)-I(x,y)\right]}^{2}\right\},$$

where I(x,y) was a gray-level intensity of the pixel at (x,y).

The Tenenbaum gradient (Tenengrad) [14] was a gradient magnitude maximization algorithm that calculated the sum of the squared values of the horizontal and vertical Sobel operators:

(2) $$f(I)=\sum_{x}\sum_{y}\left\{S_{x}{\left(x,y\right)}^{2}+S_{y}{\left(x,y\right)}^{2}\right\},$$

where $S_{x}(x,y)$ and $S_{y}(x,y)$ were the horizontal and vertical Sobel operators.

The normalized variance quantified the differences in the pixel values and the mean pixel value:

(3) $$f(I)=\frac{1}{\mu }\sum_{x,y}{\left(f_{x,y}-\mu \right)}^{2},$$

where μ was the mean pixel value of the image defined in Equation (4).

(4) $$\mu =\frac{1}{N}\sum_{x}\sum_{y}I(x,y).$$

**Figure 4:**
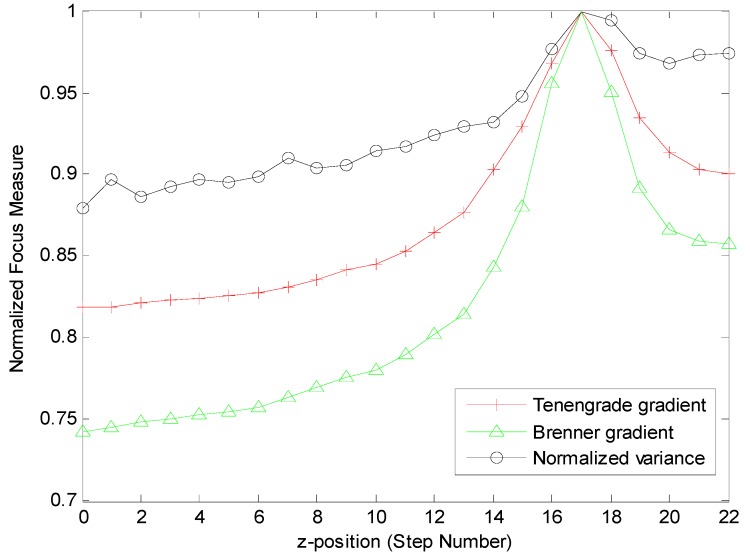
Focus curves after normalization.

With a selected focus function, the corresponding focus curve was obtained for the captured images along the complete focus interval. Figure 4 shows the normalized focus curves of each image, of which the step length is 200 μm. Different curves arrived at their global peak at the same *z*-position. All three curves correctly represented the focal plane. Some local maxima were observed with the normalized gradient function. This may prevent the autofocusing algorithm from finding the focal plane or increasing the computational complexity. When compared to the Tenengrade gradient and the normalized variance function, the Brenner function exhibited a more narrow peak, which meant good reproducibility and better searching for the focus plane.

Another evaluation criterion for the real-time visual processing system is the computational time of the focus function. A summary of the computational time required to process 23 images is presented in Table 2. The Brenner gradient function took the least time when compared to the other two functions.

Therefore, the Brenner function was chosen as the criteria function for the zebrafish embryos autofocus algorithm.

#### 3.1.2. Implementation of the Automatic Focusing Method

We used an eyepiece with a magnifying power of 10×, the objective of 4×, and a numerical aperture of 0.1. The following equation was used to calculate the depth of field:

(5) $$D_{F}=\frac{10^{-3}}{7AM}+\frac{\lambda }{2A^{2}},$$

where $D_{F}$ was the depth of field, *A* was the numerical aperture, *M* was the total magnification, λ was the light wavelength and the depth of field was *D_F_* = 63.2 μm.

**Figure 5:**
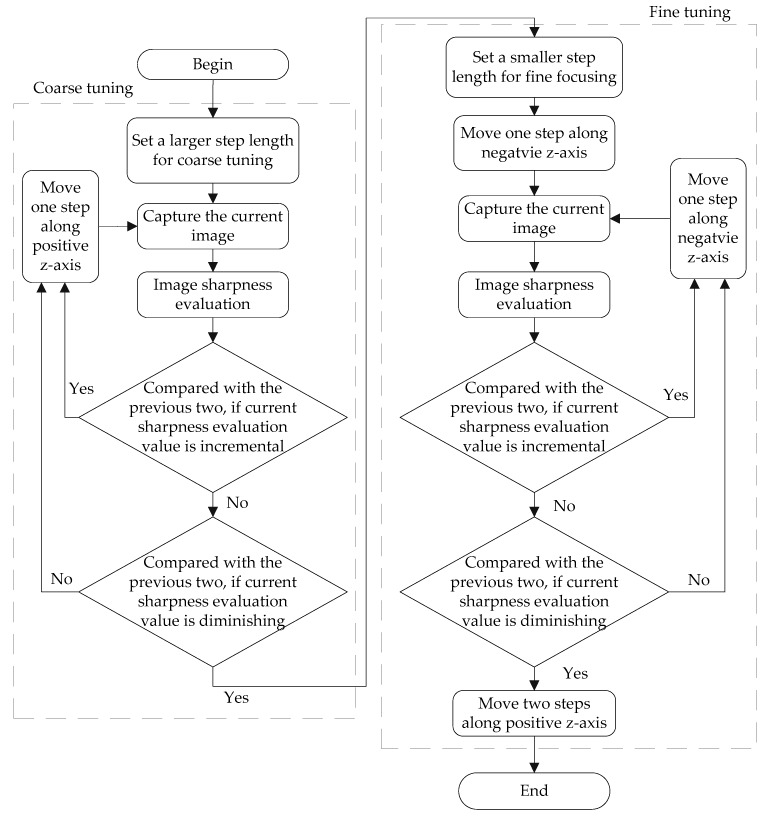
Control method schematic of the entire automatic focusing process.

If the automatic step length was larger than the depth of field, then the cells may move out of the depth of microscope field. Therefore, the step length must be smaller than $D_{F}$. To increase the speed of the focusing time, a two-phase automatic focusing method was developed, where a step length of 200 μm was used for coarse focus and 50 μm was used for fine focus. In the coarse focusing phase, the immediate sharpness evaluation value was compared with the previous two images to determine if value was incremental, which would indicate that the manipulator was moving towards the focal plane. If the sharpness evaluation value was not incremental, then it was compared with the previous two images to see if it diminished, which would indicate that the image was out-of-focus. If it was diminished, we began the fine tuning phase, in which the manipulator moved back with a step of fine tuning, similar to the coarse tuning. The control flow of the whole automatic focusing is depicted in Figure 5.

**Figure 6:**
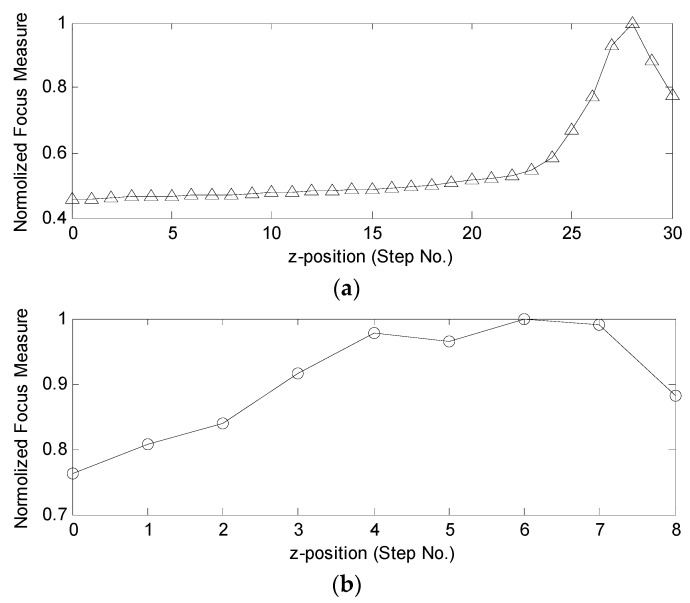
Focusing curves with Brenner gradient function: (**a**) coarse focusing with a step length of 200 μm; and (**b**) fine focusing with a step length of 50 μm.

With the Brenner focus function, the corresponding sharpness evaluation value was obtained for the captured images along the complete focus interval. Figure 6a is the coarse focusing curve with a length of 200 μm after normalization. The curve peaked at step 28, which meant it was close to the focal plane. Next, we used a fine focus at step 30 that was also marked as step 0 in the fine focusing stage. The fine focusing curve arrived at a global peak at step 6 that indicated the location of the focal plane, as shown in Figure 6b. The computational time for the Brenner gradient function to process the 31 images in coarse focusing was 0.92786 s, while the time for nine images in fine focusing was 0.23155 s.

### 3.2. Adaptive Image Processing Algorithm for Automatic Cell and Pipette Detection

This section provides real-time location information about the embryo and the injection pipette for the automatic microinjection system. The tasks include (a) detecting and locating the embryo; (b) detecting and locating the injection pipette; and (c) automatically moving the injection pipette to the center of yolk under visual servo. In a real-time automatic cell microinjecting system, one of the primary challenges is the quality of the images affected by the environment (*i.e.*, uneven illumination), cell culture medium or cell morphology. Our algorithm focused on adaptive and robust image processing.

#### 3.2.1. Real Time Adaptive-Threshold Detection for Automatic Cell Detection

**Figure 7:**
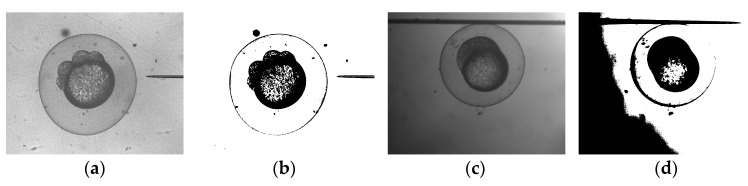
Results of conventional threshold: even illumination (**left**); uneven illumination (**right**). (**a**) Original image (even illumination); (**b**) Binary image with Otsu’s method (even illumination); (**c**) Original image (uneven illumination); (**d**) Binary image with Otsu’s method (uneven illumination).

The binary operation is a classical threshold segmentation method to separate objects of interest from the background. A conventional binary operation method uses a constant threshold *T* throughout the whole image. Some methods have been proposed to automatically calculate the value, such as the Mean Technique [15], the P-Tile Method [16], the Iterative Threshold Selection [17] and Otsu’s method [18]. Figure 7 illustrates the binary operation results using Otsu’s method. The conventional threshold was efficient for the uniform illumination images but was not ideal when the illumination was uneven.

The images were real-time video images in the automatic injection experiments, so the shadows or the direction of illumination may cause uneven illumination. Non-adaptive methods that analyze the histogram of the entire image are unsuitable. An adaptive threshold calculating method is proposed to specify an adaptive threshold for every pixel in an image. We defined the adaptive threshold as:

(6) $$T_{ij}=A_{ij}-param1,$$

where *A* was the weighted average gray value of pixels in the region around a particular pixel. The block size of the region was represented by parameters of *b* and *param*1.

**Figure 8:**
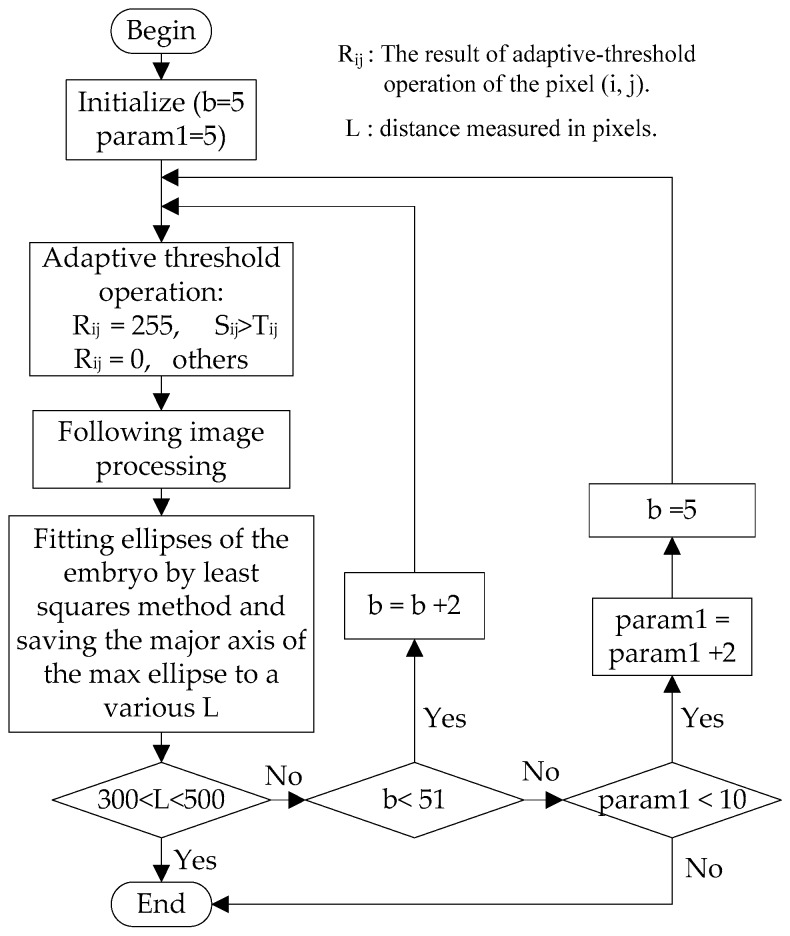
Flow diagram of parameter circulation.

In this algorithm, pixels with gray value *S_ij_* larger than their threshold *T_ij_* were set to 255, and all others were set to 0. A circulation for the two parameters was used to adjust the threshold *T_ij_* to segment the cell membrane, yolk and background from uneven illumination images. The flow diagram of the circulation to optimize the two parameters is shown in Figure 8. After the adaptive threshold obtained, a regular least squared based ellipse fitting method [19] was used to find the embryo. The contours of the image are detected and every contour is saved in the form of pixel coordinates’ vectors of the points. Then, the points in every contour are fitted to the ellipse, which is computed by minimizing the sum of the squares of the distances of the given points to an ellipse. Then, the length of the major axis of the fitted ellipse, *L*, was used to tell if the identified threshold *T_ij_* is suitable.

The adaptive-threshold detection method processed different video images in real-time and had good adaptability in both images with uniform illumination and uneven illumination, as shown in Table 3. If the image has more uneven illumination, a bigger block size is required to determine the ellipse (embryo). A green oval was used to mark the embryonic membrane, and a red dot was used to mark the embryo center. The results of our experiments showed that the proposed method can effectively detect edge of the embryo and adaptively locate the embryo center.

#### 3.2.2. Detection of Injection Pipette Tip

For a robotic microinjection system, the pipette is fixed on the robot arm. The orientation of the pipette is consequently fixed. The cell microinjection pipette has rigid-bodies with insignificant changes in size and shape along with its movement under the camera. However, the injection pipettes are usually fabricated by a micropipette puller. Even with the same setting parameters as the puller, the size of the pipette may change in a very small scale (*i.e.,* less than 1 μm). We therefore developed an optimized cross-correlation template matching algorithm to track the location of the injection pipette.

First, the tip of the injection pipette was selected as a template g[k,l], and its instance containing the object of interest was detected in a real image f[i,j]. We then measured the dissimilarity between the intensity values of the two pictures *e* as defined by a Sum-of-Squared-Deviations (SSD) template matching:

(7) $$e=\sum_{[i,j]\in R}{\left(f-g\right)}^{2}.$$

In order to reduce computational cost, Equation (7) was simplified as:

(8) $$\sum_{[i,j]\in R}{\left(f-g\right)}^{2}=\sum_{[i,j]\in R}f^{2}+\sum_{[i,j]\in R}g^{2}-2\sum_{[i,j]\in R}f\times g.$$

If *f* and *g* are fixed, ∑f×g measures a mismatch. Therefore, for an m×n template, we used

(9) $$M[i,j]=\sum_{k=1}^{m}\sum_{l=1}^{n}g[k,l]f[i+k,j+l].$$

where *k* and *l* were the displacements with respect to the templates in the image. For the automatic cell injection, the value *f* was acquired from the real-time image, which varied from the illumination environment change. To solve this problem, match measure *M* was optimized as:

(10) $$M[i,j]=\frac{\sum_{k=1}^{m}\sum_{l=1}^{n}g[k,l]f[i+k,j+l]}{{\left\{\sum {}_{k=1}^{m}\sum {}_{l=1}^{n}f^{2}[i+k,j+l]\right\}}^{1/2}}.$$

We calculated the beginning of the image coordinate [1, 1] to find the value of *M*1, and then calculated the value of the next coordinate [1, 2] as *M*2. We compare these two values, and recorded the larger value in *M*1 and recorded the coordinate of the larger value in *T*_1_[*i*, *j*]. The entire image was searched to find the largest *M* value. The corresponding coordinate *T*[*i*, *j*] was the location of successful matching.

**Figure 9:**
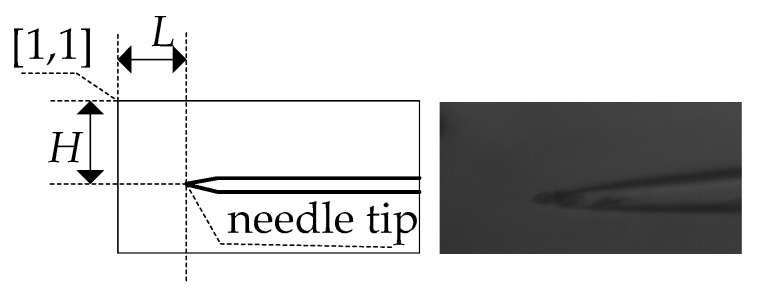
Template structure.

The coordinate [1, 1] in the template maps to the coordinate *T[i*, *j*] in the object image using this template matching algorithm, but the location of the needle tip was still unknown. Therefore, we developed a special template including the relative location of the needle tip, as shown in Figure 9. Then, we could calculate the coordinate of needle tip [*x*, *y*], where *x = i + L, y = j + H*. Thus, the precise position of the tip in a real-time image was located.

**Figure 10:**
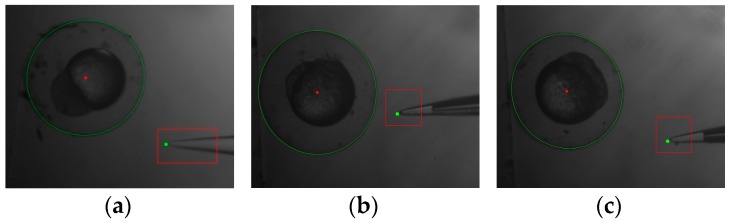
The results of image processing. (**a**) Embryo 1; (**b**) Embryo 2; (**c**) Embryo 3.

The results of the image processing are shown in Figure 10. A red rectangle was drawn to mark the region of pipette template, and a green dot was used to mark the tip of the injection pipette.

## 4. Experiments

### 4.1. Materials

**Figure 11:**
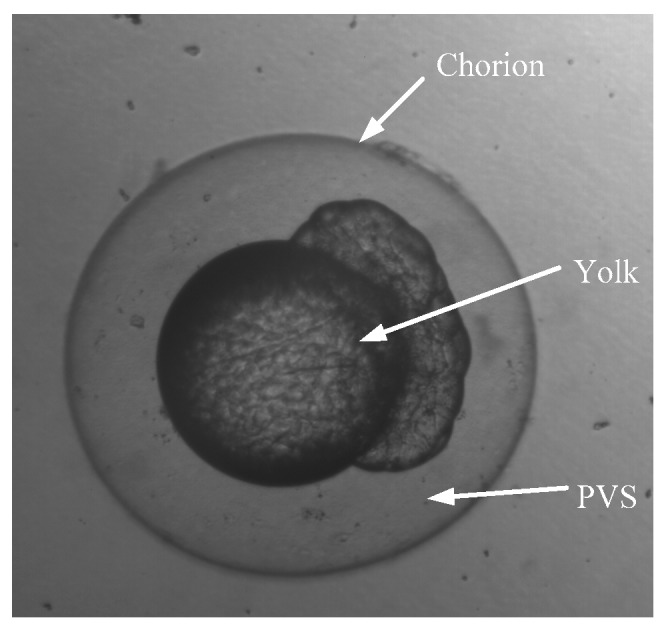
Structure of zebrafish embryo.

The zebrafish embryos were used in the visual servoing cell microinjection experiments, which were grown and collected according to the procedures described in [20]. As shown in Figure 11, the zebrafish embryo was 600–700 μm (without chorion) or 1.15–1.25 mm (with chorion) in diameter, with the cytoplasm and nucleus at the animal pole sitting upon a large mass of yolk. Various chemical substances were released during fertilization, which formed an extracellular space called the perivitelline space (PVS). The injection pipettes were fabricated by a micropipette puller (P2000, Sutter Inc.). The different diameters of the pipette tip were obtained by setting the parameters of laser heating time. Here, the pipettes with tip diameters of 20 μm were selected.

### 4.2. Experiments

Fourteen visual servoed microinjection experiments were conducted to verify the effectiveness of our developed methods. For each embryo, the injection process was as follows:

1. the petri dish containing the embryos and culture medium was placed under the microscope;
2. the embryo was autofocused by using the autofocusing algorithm;
3. the injection pipette was moved to the focus plane;
4. the adaptive image processing was used to get the location and dimension information of the embryo;
5. the template matching algorithm was used to obtain the location of the pipette tip;
6. the distance between the center of the cell and pipette tip along the *x*-axis and *y*-axis was calculated;
7. the injection pipette was automatically moved into center of the embryo;
8. the sample was deposited into the yolk section of the embryo;
9. the pipette out moved of the embryo.

**Figure 12:**
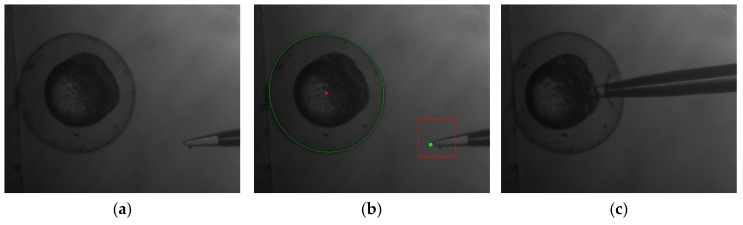
Images of automatic microinjection procedure: (**a**) embryo after autofocus; (**b**) detection of the embryo and pipette tip; and (**c**) embryo after injection.

Figure 12 and Video S1 show the typical visual servoing procedures of the microinjection experiments toward the zebrafish embryo. Figure 12a is the image of an embryo after autofocus; Figure 12b shows the successful detection of the embryo and the pipette tip, and Figure 12c is the image of the embryo after automatic injection.

Table 4 shows the results of the automatic microinjection experiments. Every embryo was successfully autofocused using the autofocus algorithm. The major and minor axis lengths show the morphology of the embryo. The second column shows parameters *b* and *param*1 for embryo detection and location. The number of block sizes affects the image processing time since more circulation is needed. With visual processing, the position information was provided for the robotic arm. All fourteen embryos were successfully injected.

## 5. Conclusions

In conventional cell injection, a manual micromanipulation procedure is conducted, which is time-consuming for high throughput and lacks reproducibility. Recently, some semi-automated cell microinjection systems were reported, but they lack robustness and partly rely on human involvement. In our research experiment, we proposed the reduction of human involvement by developing an efficient and adaptive image processing algorithm. Fourteen zebrafish embryos were injected in our experiment, which demonstrated that our system was capable of automatically injecting embryos with a success embryo recognition rate of 100%, and all of the embryos were successfully injected. However, one issue worth noting is that the computational time for the algorithm of adaptively detecting and locating embryos is a bit high. Since the parameters in the adaptive image processing algorithm can be optimized by setting more suitable initial values and developing a more time-saving looping mechanism, the manipulation time can be further reduced.

We designed and used a microrobotic cell manipulation system with an adaptive visual servoing method. We used the Brenner focus algorithm as a criteria function for cell autofocusing manipulation. We also developed an adaptive threshold tuning algorithm for automatic cell microinjection. The cell microinjection system had a 100% success rate using our adaptive imaging processing and microrobotic manipulation control. Future research will develop a knowledge-based automatic cell manipulation method in a complex environment by using deep learning.

## Figures and Tables

**Table 1:** The button functions of the user interface.

Buttons	Functions
Start Live	Open the camera, and display live video in the picture box
Stop Live	Stop video and display current image in the picture box
Save Image	Save an image to the specified location
Cell Autofocus	Begin automatic cell autofocusing manipulation
Image Process	Begin to search the cell and micropipette by visual processing algorithm and show results in the picture box and corresponding info blocks
Cell Auto Microinject	Begin to automatically move the micropipette and conduct microinjection
Exit	Save and exit the program

**Table 2:** Computational time for three selected focus functions.

Functions	Tenengrade Gradient Function	Brenner Gradient Function	Normalized Variance Function
Computational Times (s)	1.2794	0.69279	1.0831

**Table 3:** Image processing of real-time video images.

Adaptive Threshold	Cell Detection an Location	Center of Embryo	(*b*, *param*1)	Characteristic
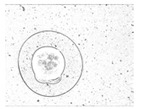	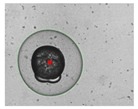	(445, 589)	(7, 7)	even illumination
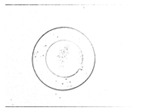	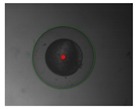	(583, 538)	(15, 5)	uneven illumination
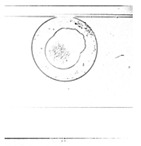	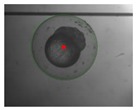	(594, 429)	(13, 5)	uneven illumination, with interference of the image of glass slice
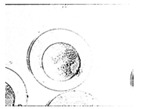	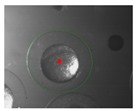	(549, 556)	(19, 5)	uneven illumination, with interference of the image of another embryo

**Table 4:** Results of the automatic microinjection.

Embryo No.	Visual Servoed Autofocus: Success	Embryo and Injection Pipette Detect and Locate			Position Information for Robot Arm		
		The Major and Minor Axis of Embryo (pixel)	Visual Processing with Adaptive-Threshold Algorithm				
			Adaptive-Threshold Parameters (*b*, *param*1)	Consumption Time (s)	Target Location of Embryo Center (pixel)	Injection Tip Location (pixel)	Success
1	√	(712, 626)	(37, 5)	2.086	(488, 513)	(969, 247)	√
2	√	(726, 636)	(47, 5)	2.824	(326, 442)	(760, 780)	√
3	√	(662, 640)	(37, 5)	2.064	(448, 407)	(989, 779)	√
4	√	(648, 604)	(45, 5)	2.673	(356, 569)	(917, 677)	√
5	√	(672, 646)	(45, 5)	2.651	(407, 590)	(815, 660)	√
6	√	(694, 660)	(25, 5)	1.149	(370, 498)	(817, 608)	√
7	√	(678, 632)	(21, 5)	0.827	(451, 528)	(884, 483)	√
8	√	(648, 614)	(29, 5)	1.482	(393, 481)	(956, 761)	√
9	√	(620, 604)	(29, 5)	1.450	(424, 541)	(903, 210)	√
10	√	(662, 624)	(35, 5)	1.903	(460, 368)	(1014, 649)	√
11	√	(666, 624)	(47, 5)	2.827	(448, 461)	(913, 200)	√
12	√	(652, 616)	(35, 5)	1.903	(477, 437)	(893, 280)	√
13	√	(648, 602)	(31, 5)	1.619	(391, 459)	(928, 697)	√
14	√	(650, 628)	(23, 5)	1.032	(564, 618)	(991, 431)	√

## Supplementary Materials

The following are available online at http://www.mdpi.com/2072-666X/7/6/104/s1, Video S1: Automatic microinjection.
